# The Prognostic Value of Arterial Stiffness According to Socioeconomic Status

**DOI:** 10.3390/jcm12216943

**Published:** 2023-11-06

**Authors:** Woo-Hyun Lim, Hack-Lyoung Kim, Hyun Sung Joh, Jae-Bin Seo, Sang-Hyun Kim, Joo-Hee Zo, Myung-A Kim

**Affiliations:** Division of Cardiology, Department of Internal Medicine, Boramae Medical Center, Seoul National University College of Medicine, Seoul 07061, Republic of Korea; woosion@gmail.com (W.-H.L.); wingx4@naver.com (H.S.J.); cetuximab@naver.com (J.-B.S.); shkimmd@snu.ac.kr (S.-H.K.); jooheezo@hanmail.net (J.-H.Z.); kma@snu.ac.kr (M.-A.K.)

**Keywords:** arterial stiffness, brachial-ankle pulse wave velocity, medical aid, national health insurance, prognosis, socioeconomic status

## Abstract

Background: Individuals of low socioeconomic status (SES) often exhibit increased cardiovascular risk factors and a worse prognosis. We conducted this study to ascertain whether brachial-ankle pulse wave velocity (baPWV), a straightforward and reliable measure of arterial stiffness, can hold prognostic value for people with low SES. Methods: We retrospectively analyzed a total of 1266 subjects (mean age 64.6 ± 11.6 years; 47.2% female) without documented cardiovascular disease who had undergone baPWV measurement. The subjects included 633 National Health Insurance Beneficiaries (NHIB) and 633 Medical Aid Beneficiaries (MAB), matched for major clinical features through a 1:1 propensity score matching method. Major adverse cardiovascular events (MACE), such as death, non-fatal myocardial infarction, non-fatal ischemic stroke, coronary revascularization, and heart failure necessitating admission, were assessed during the clinical follow-up. Results: During a median follow-up period of 4.2 years (interquartile range, 2.2–5.7 years), there were 77 MACE cases (6.1%). In multivariable Cox regression analyses, baPWV was identified as a significant predictor of MACE in both groups, regardless of the use of three different baPWV criteria (median value, Asian consensus recommendation, and cut-off value obtained by receiver operating characteristic [ROC] curve analysis). In both groups, the baPWV value obtained using ROC curve analysis emerged as the best predictor of MACE. This predictive value was stronger in the NHIB group (hazard ratio, 5.80; 95% confidence interval, 2.30–14.65; *p* < 0.001) than in the MAB group (hazard ratio, 3.30; 95% confidence interval, 1.57–6.92; *p* = 0.002). Conclusions: baPWV was associated with future MACE incidence in both NHIB and MAB groups. Since baPWV is simple and cost-effective to measure, it could be efficiently used as a risk stratification tool for individuals with low SES.

## 1. Introduction

Over time, the elasticity of arterial walls decreases, a natural process accentuated by age. Chronic exposure to certain risk factors accelerates this stiffening. Notable among these risks are high blood pressure, dyslipidemia, elevated blood sugar levels or hyperglycemia, tobacco consumption, and persistent inflammation [1,2].

The stiffening of the arterial wall is a complex physiological change with significant clinical implications. When arteries become rigid, they lose their ability to adequately buffer the pulsatile energy originating from the heart’s contractions. This energy, instead of being absorbed, gets transmitted onward, which can ultimately lead to damage to vital organs [3]. These phenomena highlight the essential importance of understanding arterial stiffness in a clinical context. In particular, arterial stiffness stands out in that it can independently predict the cardiovascular risk an individual may face, setting it apart from other traditional risk factors. Over the past few decades, many researchers have conducted numerous studies illuminating the prognostic value of arterial stiffness. These studies, encompassing diverse patient groups as well as the broader general population, consistently highlight its undeniable importance in predicting cardiovascular outcomes [4,5].

Understanding the complex interplay between socioeconomic status (SES) and cardiovascular outcomes is imperative in modern healthcare. Individuals with a lower SES consistently demonstrate a heightened risk of cardiovascular diseases [6,7]. This elevated risk can be attributed to several factors, including increased exposure to detrimental health practices such as poor dietary habits, sedentary lifestyles, tobacco consumption, and excessive alcohol intake. Further exacerbating the situation, those in the lower SES often encounter barriers to accessing quality healthcare, hindering early detection and intervention [8,9]. Additionally, the stress associated with financial insecurity and inadequate living conditions can contribute to hypertension and other heart-related conditions [10]. As a result, there is a critical need for proactive risk assessment and comprehensive, enhanced treatment approaches for patients with low SES. Recognizing these disparities and formulating strategies tailored to this demographic can potentially bridge the health inequality gap, ensuring that cardiovascular care is equitable and efficient.

Up until now, there hasn’t been an adequately proposed technique that serves as an effective risk stratification tool specifically tailored for patients with low SES when we set aside the conventional risk indicators. Our research team postulated that the brachial-ankle pulse wave velocity (baPWV) might offer a promising solution in this domain. This is especially due to its attributes of being relatively straightforward and cost-effective to measure. If baPWV demonstrates significant prognostic value for low-SES individuals, it may aid in risk assessment for this population. In light of this, the primary objective of our research is to meticulously evaluate the prognostic significance of baPWV concerning SES. We are particularly interested in assessing its potential to predict outcomes in patients with low SES, which, if proven, could be an invaluable tool in cardiovascular risk prediction.

## 2. Methods

### 2.1. Study Subjects and Design

The specific methods for this study have already been published [11]. This research was carried out as a retrospective analysis at Boramae Medical Center, a general hospital situated in Seoul, the capital of the Republic of Korea. The framework of this study was anchored in a single-center approach to ensure consistency in data collection and analysis. Spanning a period from January 2010 to December 2016, we meticulously screened individuals aged between 20 and 90 who visited the cardiovascular center of Boramae Medical Center. The participants selected for this study were those who had no prior documented instances of cardiovascular diseases. Upon their visit, these individuals underwent a baPWV test, a standard procedure at Boramae Medical Center. This test is an integral part of the hospital’s routine diagnostic toolkit, specifically utilized to forecast potential cardiovascular risks an individual might face. The responsibility of administering this test typically rests with the seasoned attending physician, ensuring precision and accuracy in the procedure. The choice of Boramae Medical Center as this study location also underscores its reputation as a trusted medical institution in a major urban setting, thus providing a diverse patient demographic for this study. Patients were excluded if they had any of the following conditions: (1) unstable vital signs; (2) recently worsened chest pain or dyspnea; (3) resting dyspnea or chest pain; (4) a left ventricular ejection fraction of less than 50%; (5) valvular stenosis or regurgitation exceeding a mild degree; (6) the presence of pericardial effusion or congenital heart defect; (7) uncontrolled cardiac arrhythmia; and (8) an ankle-brachial index less than 0.9 or greater than 1.4. This study protocol was approved by the Institutional Review Board (IRB) of Boramae Medical Center (IRB number, 30-2023-21). Informed consent was waived by the IRB due to the routine nature of the data collected and this study’s retrospective design.

### 2.2. National Health Insurance vs. Medical Aid Beneficiaries

The healthcare system in the Republic of Korea combines compulsory National Health Insurance (NHI) and private healthcare services. The NHI, covering approximately 97% of the population, operates as a single-payer system financed by mandatory contributions from all residents, dependent on income and assets [12]. This system delivers a comprehensive range of medical services, including inpatient, outpatient, and preventive care. Medical Aid (MA) in the Republic of Korea is a public assistance program designed to offer free medical services to low-income individuals and families who cannot afford health insurance [13]. The program is managed by the Ministry of Health and Welfare and is funded through national and local taxes. Eligibility for MA is ascertained through a means test, assessing income, assets, and the potential earning capacity of the individual and their family. Eligible MA recipients are classified as either Type 1 or Type 2. Type 1 recipients include those incapable of labor, such as the elderly, disabled, or children under 18, while Type 2 recipients are able-bodied but currently unemployed individuals. It was reported that MA beneficiaries (MAB) had low levels of education (≤elementary: 60.9% vs. 39.9%) and household income (low ordinary income: 94.8% vs. 33.6%), and the proportion of those with no economic activity was significantly higher (81.7% vs. 57.1%), compared with NHI beneficiaries (NHIB) [14]. MAB accounted for 2.9% of all Koreans in 2017, while other Koreans (97.1%) were NHIB [15]. Given that this study was conducted at a metropolitan government hospital, the proportion of MAB, at 7.8%, was considerably higher than the average.

### 2.3. Clinical Data Collection

The process of measuring blood pressure in participants was executed utilizing an oscillometric device, a standard instrument known for its precision in clinical settings. In order to determine the body mass index (BMI) of each individual, the formula employed involved dividing the person’s body weight, measured in kilograms (kg), by the square of their height, expressed in meters (m). For the classification of diabetes mellitus, several criteria were considered: a prior diagnosis from a medical professional, the current administration of anti-diabetic medications, fasting blood glucose levels equal to or exceeding 126 mg/dL, or a glycated hemoglobin reading that is equal to or surpasses 6.5%. The criteria for identifying hypertension in individuals included a prior diagnosis confirmed by a medical practitioner, the ongoing use of anti-hypertensive drugs, recorded systolic blood pressure readings of 140 mmHg or more, or diastolic blood pressure readings equal to or above 90 mmHg. Dyslipidemia, on the other hand, was recognized in individuals either consuming anti-dyslipidemic medications at the time or those who had a level of low-density lipoprotein cholesterol that was 160 mg/dL or higher. Regarding the assessment of smoking habits, any individual who had been consistently smoking within the preceding year was categorized as a smoker. Furthermore, a comprehensive collection of data was undertaken to ascertain the use of various medications by participants. This encompassed a diverse range of pharmaceuticals, from statins and calcium channel blockers to beta-blockers and agents that modulate the renin-angiotensin system. The objective behind this meticulous documentation was to ensure a holistic understanding of each participant’s health profile.

### 2.4. baPWV Measurement

The methods for baPWV measurement were previously described [16,17]. The measurement of brachial-ankle pulse wave velocity (baPWV) was conducted using the volume-plethysmography device VP-1000 (Colins, Komaki, Japan). On the day of the examination, the consumption of alcohol, cigarette smoking, and the intake of caffeine-containing beverages were prohibited, though the continuation of regular medications was permitted. Prior to the assessment, the patient was positioned in a calm and secluded area, where they were asked to lie down and rest for a minimum of 5 min. The measurement process involves placing blood pressure cuffs equipped with sensors around the patient’s arms and ankles. The cuffs are connected to an oscillometric device. When the heart contracts, it sends a pulse wave through the arteries. The sensors in the cuffs detect the pulse wave as it arrives at the brachial artery (in the arm) and at the tibial artery (near the ankle). The time it takes for the wave to travel from the brachial artery to the tibial artery, known as the pulse transit time, is recorded. The distance between the brachial and tibial arteries is estimated using a formula that takes into account the patient’s height. The pulse wave velocity is then calculated by dividing this distance by the pulse transit time.

### 2.5. Clinical Outcome

The core focal point of this comprehensive research was to identify the occurrence of major adverse cardiovascular events (MACE). The MACE included a range of significant cardiovascular episodes, including all-cause mortality, incidents of non-fatal myocardial infarctions, procedures related to coronary revascularization, episodes of non-fatal ischemic strokes, and scenarios where heart failure was so pronounced that it necessitated immediate hospital admission. Delving into specifics, a myocardial infarction, a critical cardiac event, was meticulously characterized. Its identification hinged upon a constellation of symptoms and clinical findings: the patient experiencing chest pain, a notable elevation in troponin levels, observable alterations in electrocardiograms, and any pertinent revelations emerging from invasive coronary angiography. Meanwhile, the term “coronary revascularization” was employed to denote a suite of interventional procedures designed to restore optimal blood flow to the heart muscles. This encompassed both percutaneous coronary interventions, which are minimally invasive procedures to open up blocked coronary arteries, and more elaborate coronary artery bypass surgeries, where grafts are used to redirect blood around blocked arteries. Transitioning to ischemic strokes, these were discerned when an individual exhibited an abrupt onset of neurological deficits. Such clinical signs had to be confirmed by corresponding abnormalities found in brain imaging studies such as computed tomography or magnetic resonance imaging. Heart failure as a clinical event was adjudicated when, upon a patient’s hospital discharge, the primary diagnosis revolved around heart failure. In situations where participants experienced a series of clinical events, the analytical focus was directed to the first episode, recognizing it as the defining clinical event for that individual.

### 2.6. Statistical Analysis

Numbers are expressed as mean ± standard deviation for continuous variables and *n* (%) for categorical variables. The Chi-square test and the Student’s *t*-test were used for categorical and continuous variables, respectively, in the comparison between the NHIB and MAB groups. Propensity score matching was performed to achieve a 1:1 balance between the NHIB and MAB groups, predicated on the following clinical variables: sex, age, systolic blood pressure, body mass index, the presence of diabetes mellitus, hypertension, dyslipidemia, current smoking habits, and the usage of specific medications, namely statins, calcium channel blockers, beta-blockers, and renin-angiotensin system blockers. In multivariable analyses, baPWV was converted into a dichotomous variable according to the following three criteria: (1) baPWV ≥ 1800 cm/s, based on the high-risk patient criterion suggested by the recent Asian consensus [18], (2) baPWV ≥ 1633 cm/s for the NHIB group and baPWV ≥ 1888 cm/s for the MAB group, based on median values; and (3) baPWV ≥ 1734 cm/s for the NHIB group and baPWV ≥ 1811 cm/s for the MAB group, as a cut-off value derived from receiver operating characteristic curve (ROC) analysis. Each multivariate Cox analysis was performed using these three baPWV values. The C-index was calculated to compare the prognostic value of each baPWV cut-off value. The following potential confounders were controlled for during each multivariable analysis: age, sex, hypertension, diabetes mellitus, and current smoking. Kaplan–Meier survival curve analysis was performed to assess the difference in the MACE-free survival rate between NHIB and MAB using a log-rank test. A *p* value of < 0.05 was used to indicate statistical significance. All statistical tests were performed with SPSS for Windows version 22 (IBM Co., Armonk, NY, USA) and R version 4.3.1 (R Foundation for Statistical Computing, Vienna, Austria).

## 3. Results

**Figure 1:**
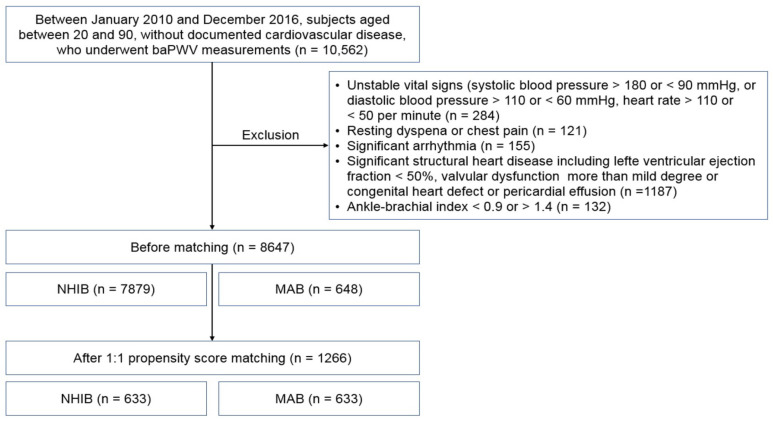
The flow chart for study enrollment: baPWV, brachial-ankle pulse wave velocity; NHIB, national health insurance beneficiaries; MAB, medical aid beneficiaries.

**Figure 2:**
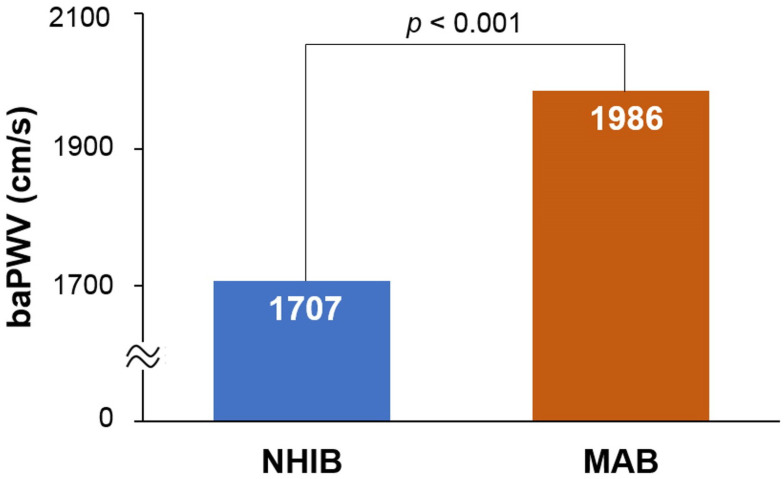
The difference in baPWV between the NHIB and MAB groups: baPWV, brachial-ankle pulse wave velocity; NHIB, national health insurance beneficiaries; MAB, medical aid beneficiaries.

The flow chart for study enrollment is shown in Figure 1. Baseline clinical characteristics of this study population, both before and after 1:1 propensity score matching, are shown in Table 1. Before matching, a total of 8647 subjects were analyzed, comprised of 7879 subjects in the NHIB group and 648 in the MAB group. Beneficiaries in the MAB group were older than those in the NHIB group, with average ages of 64.7 ± 12.2 years and 59.9 ± 12.4 years, respectively (*p* < 0.001). The MAB group had higher systolic blood pressure and a greater number of risk factors, including diabetes mellitus, hypertension, and cigarette smoking, compared to the NHIB group (*p* < 0.05 for each). The NHIB group had more prescriptions for calcium channel blockers and RAS blockers, whereas beta-blockers were more commonly prescribed in the MAB group. When 1:1 matching was performed with 633 subjects in each group, the differences in these clinical factors between the two groups were eliminated. The clinical characteristics of this study subjects after matching are also detailed in Table 1. In the matched set of study subjects (*n* = 1266), the MAB group had a significantly higher baPWV value than the NHIB group (1985 ± 496 vs. 1706 ± 385 cm/s; *p* < 0.001) (Figure 2).

The clinical outcomes of the matched set of study subjects (*n* = 1266) are outlined in Table 2. During a median follow-up period of 4.2 years (interquartile range, 2.2–5.7 years), there were 77 cases of MACE (6.1%), which included 30 deaths (2.4%), 5 AMI (0.4%), 34 revascularizations (2.7%), 13 ischemic strokes (1.0%), and 9 heart failures (0.9%). Although numerically, there appeared to be a higher incidence of MACE and each individual clinical event in the MAB group compared to the NHIB group, the difference was not statistically significant.

**Figure 3:**
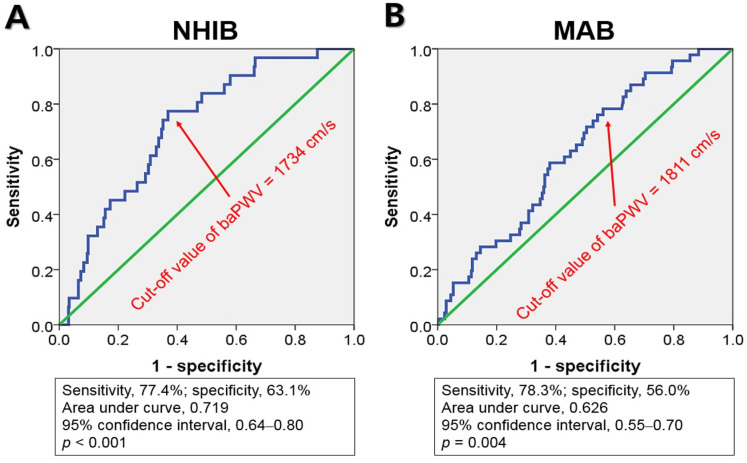
Receiver operating characteristic curve analyses show the cut-off values of baPWV, NHIB, and MAB groups: baPWV, brachial-ankle pulse wave velocity; NHIB, national health insurance beneficiaries (**A**); MAB, medical aid beneficiaries (**B**).

ROC curve analysis indicated that the cut-off values of baPWV for predicting MACE were 1734 cm/s for NHIB and 1811 cm/s for MAB, respectively (Figure 3).

**Figure 4:**
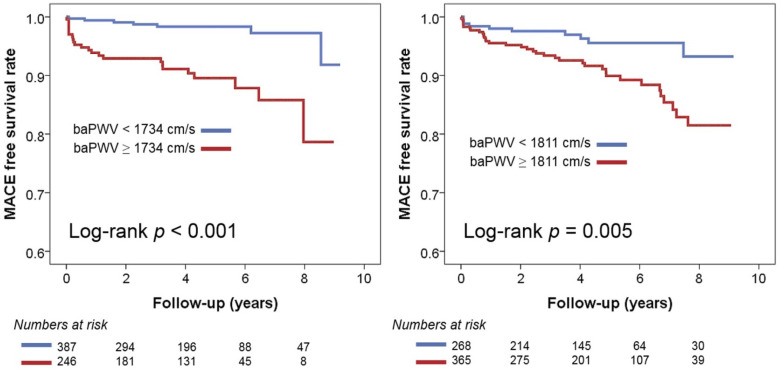
Kaplan–Meier survival curves showing the prognostic value of baPWV in the NHIB and MAB groups: baPWV, brachial-ankle pulse wave velocity; NHIB, national health insurance beneficiaries; MAB, medical aid beneficiaries.

In multivariable Cox regression analyses, baPWV was identified as a significant predictor of MACE in both groups, regardless of the use of three different baPWV criteria (Table 3). In the recent Asia–Pacific expert statement, a baPWV > 1800 cm was proposed as a high-risk criterion [18]. This increases the risk of MACE by 3.12 times (95% confidence interval [CI], 1.41–6.90; *p* = 0.005) in NHIB and 3.19 times (95% CI, 1.52–6.73; *p* = 0.002) in MAB. When the risk classification was determined based on the median value of baPWV, having a baPWV higher than the median was associated with a 4.4-fold increase (95% CI, 1.67–11.58; *p* = 0.003) in the occurrence of MACE in the NHIB group and a 2.31-fold increase (95% CI, 1.19–4.46; *p* = 0.013) in the MAB group. In both groups, the baPWV value obtained using ROC curve analysis emerged as the best predictor of MACE. This predictive value was stronger in the NHIB group (hazard ratio, 5.80; 95% CI, 2.30–14.65; *p* < 0.001) compared to the MAB group (hazard ratio, 3.30; 95% CI, 1.57–6.92; *p* = 0.002). The C-index value for the baPWV cut-off was higher using ROC curve analysis compared to the other two cut-off values, but there was no statistically significant difference. Figure 4 presents the results of the Kaplan–Meier analysis on the MACE prediction in each group using the cut-off value of baPWV derived from the ROC curve analysis.

## 4. Discussion

We conducted a 1:1 match with the NHIB group to assess whether baPWV holds prognostic value in the MAB group, typically characterized by low SES. Our findings revealed that the prognostic value of baPWV for predicting MACE was significant in both groups. To the best of our knowledge, this is the first study to demonstrate the prognostic value of baPWV in relation to SES.

The dynamics of arterial stiffness and its impact on cardiovascular health remain a topic of significant medical interest. One of the paramount observations in cardiovascular physiology is the direct relationship between elevated arterial stiffness and a concurrent surge in systolic pressure. This increase in systolic pressure becomes a precursor for conditions like left ventricular hypertrophy. Conversely, when diastolic pressure drops, it results in diminished coronary blood flow, a factor of grave clinical concern [1,2,3]. Parallel to this, there’s an observable augmentation in the prevalence of cardiovascular risk determinants that coincide with elevated arterial stiffness. This coexistence further escalates the patient’s vulnerability to cardiovascular complications [2]. Based on these factors, it is well established that increased arterial stiffness predicts future cardiovascular events or deaths, not only in various patient groups but also in the general population [4,5].

In cardiovascular research, certain gaps remain despite extensive studies. One such gap relates to how arterial stiffness and SES interact. While we know arterial stiffness can predict cardiovascular outcomes, few studies consider its value when accounting for an individual’s SES. Our research addressed this gap. In this study, we confirmed that baPWV was a significant predictor of cardiovascular events in both the NHIB group with average SES and the MAB group with low SES. These results suggest that baPWV can play an important role in assessing a patient’s risk, regardless of the patient’s SES status.

In our investigation, we specifically highlighted the significance of baPWV as a prognostic tool in patients belonging to the MAB group, which is characterized by a lower SES. We aimed to identify predictive validity through rigorous evaluation using three cut-off points for baPWV. Our findings consistently indicated that baPWV serves as a robust prognostic marker for individuals within this category. Given its methodological simplicity combined with cost efficiency, baPWV emerges as an essential diagnostic resource. This holds particular resonance for those stemming from lower SES backgrounds, as they can derive considerable advantage from such a convenient yet potent risk stratification mechanism. The potential implications of this study highlight the need for further exploration in this area, particularly when tailoring cardiovascular risk assessment strategies for different SES groups.

Increasing evidence highlights the disparities in cardiovascular health outcomes based on SES, with individuals of lower SES presenting worse cardiovascular risk profiles and prognoses [6,7]. This disparity is likely due to a myriad of factors, including limited access to healthcare, poorer lifestyle habits, higher stress levels, and the greater prevalence of other comorbidities [8,9]. In alignment with this, our results also showed that individuals from the MAB group had more cardiovascular risk factors and higher arterial stiffness levels than those from the NHIB group. Although there was no statistical significance, the incidence of cardiovascular events was numerically higher in the MAB group than in the NHIB group in our study. Given this background, the need for an accessible, simple, and cost-effective risk stratification tool for cardiovascular disease becomes paramount for people with low SES. The baPWV measurement could fill this role effectively. It is simple, non-invasive, and inexpensive [18], which makes it highly suitable for use in low-SES populations that might otherwise struggle to access more complex or costly diagnostic tools. Furthermore, the predictive value of baPWV measurements in various clinical settings has been demonstrated in numerous studies [5]. The application of baPWV as a risk stratification tool in low-SES populations could lead to earlier detection of cardiovascular risk, enabling timely intervention and potentially improving long-term health outcomes. However, further research is needed to establish baPWV as a standard risk stratification tool in these populations and to gain better insight into how socioeconomic factors may influence its predictive accuracy.

Among various methods to measure arterial stiffness, pulse wave velocity (PWV) is the most widely used. There are several types of PWV measurement methods depending on the location of the measuring arteries, of which carotid-femoral PWV (cfPWV) and baPWV are most commonly used in clinical and research areas [2,18]. Initially developed, cfPWV is deemed the gold standard for noninvasively measuring arterial stiffness [19]. This is due to its focus on the elastic aortic region, with its efficacy backed by numerous clinical studies [4]. Yet, its precision depends on accurate probe placement on the carotid and femoral arteries, necessitating expertise and possibly causing patient discomfort. It’s particularly challenging for individuals with obesity or shorter necks to pinpoint the artery’s target location [18]. Conversely, baPWV emerged later in Japan, offering a simpler, more patient-friendly approach. Its procedure, wrapping a blood pressure cuff around the upper arm and ankle, eliminates any discomfort. Furthermore, its uncomplicated nature means the tester’s proficiency doesn’t significantly impact results. However, there is some criticism regarding baPWV as a true arterial stiffness metric since it encompasses some muscular arteries. Despite this, its efficacy is confirmed by multiple validation studies [5]. On the one hand, it has been reported that baPWV represents the total arterial (central peripheral) value and therefore better represents the afterload of the heart [20]. Recently, baPWV’s use has surged, especially in Asia, attributed to its non-invasiveness and ease of measurement. It’s especially advantageous for mass screenings, aligning with contemporary healthcare goals [21].

Our study, while informative, does have several limitations due to its retrospective design. First, given the relatively small number of clinical events and subjects (*n*), it was challenging to ascertain the significance of each individual event. In other words, our study may not have had sufficient statistical power to detect differences or trends for each specific event. Second, a common limitation of retrospective studies that is also applicable here is the potential for missed clinical events. There may have been instances where clinical events occurred but were not recorded or confirmed, leading to an underestimation of the actual event rate. Third, despite our use of propensity score matching and multivariate analysis, there may have been other confounding variables that were not accounted for. These unmeasured variables might have affected our results, introducing bias into our study. Lastly, given the specific demographic and racial characteristics of our study population, it is challenging to directly extrapolate our findings to other racial or population groups. Our results may not be generalizable to other groups with different genetic, lifestyle, and socio-economic characteristics. Future studies with diverse population groups are needed to validate and extend our findings.

## 5. Conclusions

Our results demonstrate that baPWV has significant prognostic value in predicting MACE in both the general population (NHIB group) and a low SES group (MAB group). Given that the baPWV measurement is simple and cost-effective, it might be particularly beneficial for risk stratification in patients with low SES.

## Figures and Tables

**Table 1:** Baseline clinical characteristics of study population before and after 1:1 propensity score matching.

	Before Matching				After Matching			
Variable	NHIB (*n* = 7879)	MAB (*n* = 648)	SMD	*p*	NHIB (*n* = 633)	MAB (*n* = 633)	SMD	*p*
Sex			0.011	0.782			−0.048	0.409
Male	4312 (54.7)	351 (54.2)			327 (51.6)	342 (54.1)		
Female	3567 (45.3)	297 (45.8)			306 (48.4)	291 (45.9)		
Age, years	59.9 ± 12.4	64.7 ± 12.2	0.390	<0.001	64.7 ± 11.0	64.5 ± 12.2	−0.017	0.719
Systolic blood pressure, mmHg	130 ± 17	132 ± 19	0.117	0.041	132 ± 18	132 ± 19	−0.006	0.906
Body mass index, kg/m^2^	24.8 ± 3.3	24.6 ± 3.9	−0.059	0.142	24.5 ± 3.4	24.6 ± 3.9	0.027	0.609
Diabetes mellitus	1617 (20.5)	278 (42.9)	0.452	<0.001	262 (41.3)	263 (41.5)	0.003	0.935
Hypertension	3359 (42.6)	403 (62.1)	0.403	<0.001	408 (64.4)	388 (61.3)	−0.065	0.163
Dyslipidemia	1583 (20.1)	152 (23.4)	−0.096	0.051	127 (20.1)	129 (20.4)	0.017	0.745
Current smoking	824 (10.4)	136 (20.9)	0.259	<0.001	109 (17.2)	122 (19.2)	0.050	0.325
Statin	3132 (39.7)	248 (38.2)	−0.030	0.459	252 (39.8)	244 (38.5)	−0.026	0.640
Calcium channel blockers	1803 (22.8)	115 (17.7)	−0.134	0.002	118 (18.6)	114 (18.0)	−0.020	0.765
Beta-blockers	1362 (17.2)	137 (21.1)	0.094	0.013	123 (19.4)	133 (23.0)	0.039	0.468
RAS blockers	2049 (26.0)	126 (19.4)	−0.166	0.000	133 (21.0)	126 (19.9)	−0.028	0.616

Numbers are expressed as mean ± standard deviation, or *n* (%). NHIB, national health insurance beneficiaries; MAB, medical aid beneficiaries; SMD, standardized mean difference; RAS, renin-angiotensin system.

**Table 2:** Clinical events in each group of matched set.

Event	NHIB (*n* = 633)	MAB (*n* = 633)	*p*
MACE	31 (4.9)	46 (7.3)	0.078
Death	12 (1.9)	18 (2.8)	0.268
Non-fatal myocardial infarction	2 (0.3)	3 (0.5)	0.999
Coronary revascularization	16 (2.5)	18 (2.8)	0.728
Non-fatal ischemic stroke	4 (0.6)	9 (1.4)	0.163
Heart failure requiring admission	3 (0.5)	6 (0.9)	0.506

Numbers are expressed as *n* (%). MACE, major adverse cardiovascular event; NHIB, national health insurance beneficiaries; MAB, medical aid beneficiaries.

**Table 3:** Prognostic value of baPWV in each group of matched set.

Group and baPWV Value	HR (95% CI)	*p*	C-Index	*p*	
NHIB					
baPWV ≥ 1800 cm/s	3.12 (1.41–6.90)	0.005	0.70 (0.61–0.79)	Ref.	
baPWV ≥ 1633 cm/s	4.40 (1.67–11.58)	0.003	0.72 (0.64–0.79)	0.616	Ref.
baPWV ≥ 1734 cm/s	5.80 (2.30–14.65)	<0.001	0.74 (0.66–0.81)	0.088	0.334
MAB					
baPWV ≥ 1800 cm/s	3.19 (1.52–6.73)	0.002	0.67 (0.57–0.76)	Ref.	
baPWV ≥ 1888 cm/s	2.31 (1.19–4.46)	0.013	0.65 (0.55–0.75)	0.439	Ref.
baPWV ≥ 1811 cm/s	3.30 (1.57–6.92)	0.002	0.67 (0.58–0.76)	0.789	0.444

Each baPWV reference value was applied to a separate multivariable analysis model. The following potential confounders were controlled during each multivariable analysis: age, sex, hypertension, diabetes mellitus, and current smoking. baPWV, brachial-ankle pulse wave velocity; HR, hazard ratio; CI, confidence interval; NHIB, national health insurance beneficiaries; MAB, medical aid beneficiaries; Ref., reference.

## Data Availability

The datasets used and/or analyzed during the current study available from the corresponding author on reasonable request.

## References

[1] Chirinos J.A., Segers P., Hughes T., Townsend R. (2019). Large-Artery Stiffness in Health and Disease: JACC State-of-the-Art Review. J. Am. Coll. Cardiol..

[2] Cavalcante J.L., Lima J.A., Redheuil A., Al-Mallah M.H. (2011). Aortic stiffness: Current understanding and future directions. J. Am. Coll. Cardiol..

[3] Weber T. (2020). The Role of Arterial Stiffness and Central Hemodynamics in Heart Failure. Int. J. Heart Fail..

[4] Vlachopoulos C., Aznaouridis K., Stefanadis C. (2010). Prediction of cardiovascular events and all-cause mortality with arterial stiffness: A systematic review and meta-analysis. J. Am. Coll. Cardiol..

[5] Ohkuma T., Ninomiya T., Tomiyama H., Kario K., Hoshide S., Kita Y., Inoguchi T., Maeda Y., Kohara K., Tabara Y. (2017). Brachial-Ankle Pulse Wave Velocity and the Risk Prediction of Cardiovascular Disease: An Individual Participant Data Meta-Analysis. Hypertension.

[6] Stringhini S., Carmeli C., Jokela M., Avendaño M., Muennig P., Guida F., Ricceri F., d’Errico A., Barros H., Bochud M. (2017). Socioeconomic status and the 25 × 25 risk factors as determinants of premature mortality: A multicohort study and meta-analysis of 1·7 million men and women. Lancet.

[7] Signorello L.B., Cohen S.S., Williams D.R., Munro H.M., Hargreaves M.K., Blot W.J. (2014). Socioeconomic status, race, and mortality: A prospective cohort study. Am. J. Public Health.

[8] Zhang Y.B., Chen C., Pan X.F., Guo J., Li Y., Franco O.H., Liu G., Pan A. (2021). Associations of healthy lifestyle and socioeconomic status with mortality and incident cardiovascular disease: Two prospective cohort studies. BMJ.

[9] Candio P., Mujica F.P., Frew E. (2023). Socio-economic accounting of inequalities in excess weight: A population-based analysis. BMC Public Health.

[10] Spruill T.M. (2010). Chronic psychosocial stress and hypertension. Curr. Hypertens. Rep..

[11] Kim H.L., Lee J.Y., Lim W.H., Seo J.B., Kim S.H., Zo J.H., Kim M.A. (2020). Relationship of Socioeconomic Status to Arterial Stiffness: Comparison Between Medical Aid Beneficiaries and National Health Insurance Beneficiaries. Am. J. Hypertens..

[12] Jeon B., Kwon S. (2013). Effect of private health insurance on health care utilization in a universal public insurance system: A case of South Korea. Health Policy.

[13] Ahn Y.H., Kim E.S., Ham O.K., Kim S.H., Hwang S.S., Chun S.H., Gwon N.Y., Choi J.Y. (2011). Factors associated with the overuse or underuse of health care services among medical aid beneficiaries in Korea. J. Community Health Nurs..

[14] Kim J.H., Lee K.S., Yoo K.B., Park E.C. (2015). The differences in health care utilization between Medical Aid and health insurance: A longitudinal study using propensity score matching. PLoS ONE.

[15] Bahk J., Kang H.Y., Khang Y.H. (2019). Trends in life expectancy among medical aid beneficiaries and National Health Insurance beneficiaries in Korea between 2004 and 2017. BMC Public Health.

[16] Koh H.B., Ryu J.H., Kim S.S., Kim M.G., Park J.B., Kim C.D., Kang K.P., Ro H., Han S.Y., Huh K.H. (2023). Association between sclerostin levels and vascular outcomes in kidney transplantation patients. J. Nephrol..

[17] Miyagi T., Ishida A., Shinzato T., Ohya Y. (2023). Arterial Stiffness Is Associated With Small Vessel Disease Irrespective of Blood Pressure in Stroke-Free Individuals. Stroke.

[18] Park J.B., Sharman J.E., Li Y., Munakata M., Shirai K., Chen C.H., Jae S.Y., Tomiyama H., Kosuge H., Bruno R.M. (2022). Expert Consensus on the Clinical Use of Pulse Wave Velocity in Asia. Pulse.

[19] Laurent S., Cockroft J., Bortel L.V., Boutouyrie P., Giannattasio D.H., Pannier B., Vlachopoulos C., Wilkinson I., Struijker-Boudier H., European Netsork for Non-invasive Investigation of Large Arteries (2006). Expert Consensus document on arterial stiffness: Methodolgical issues and clinical applications. Eur. Heart J..

[20] Chow B., Rabkin S.W. (2013). Brachial-ankle pulse wave velocity is the only index of arterial stiffness that correlates with a mitral valve indices of diastolic dysfunction, but no index correlates with left atrial size. Cardiol. Res. Pract..

[21] Tomiyama H., Shiina K. (2020). State of the Art Review: Brachial-Ankle PWV. J. Atheroscler. Thromb..

